# Mung Bean Starch-Derived Fermented Liquid Alleviates Constipation via 5-HT Modulation and Gut Microbiota Regulation: An In Vivo Study

**DOI:** 10.3390/foods14142483

**Published:** 2025-07-16

**Authors:** Tao Ma, Mengtian Zhou, Xinru Zhang, Ruixue Zhang, Ying Wei, Jifeng Liu

**Affiliations:** 1 College of Food Science and Engineering, Tianjin University of Science and Technology, Tianjin 300457, China; 18701452049@163.com; 2 Key Food Science and Engineering College, Beijing University of Agriculture, Beijing 102206, China; 18097725169@163.com (M.Z.); 20057914@bua.edu.cn (X.Z.); 3 Key Laboratory of Agricultural Microbiome (MARA), Biotechnology Research Institute, Chinese Academy Agricultural Sciences, Beijing 100081, China; zrx19941217@163.com

**Keywords:** mung bean starch fermentation liquid, fermentation, constipation, intestinal microbiota, serotonin

## Abstract

Background: Constipation is a common gastrointestinal disorder with a significant impact on quality of life. Methods: Constipation was induced in male ICR mice via 25% cotrimoxazole gavage (20 mL/kg/day for 7 days). Mice were divided into prevention (pre-MBSFL), treatment (MBSFL), and control groups. MBSFL was prepared by fermenting mung bean starch with *Lactobacillus plantarum* (1:3 *w*/*v* ratio, 37 °C for 48 h), and administered via daily oral gavage (250 mg/kg bw) for 14 days. Fecal parameters (water content and first black stool latency), gastrointestinal motility (gastric emptying and small intestinal propulsion), serum biomarkers (NO, VIP, SP, and 5-HT), and intestinal gene expression (5HTR_4_, SERT, and MAO_A_) were analyzed. Results: MBSFL intervention restored fecal water content by 38%, reduced first black stool latency from 6.2 h to 3.1 h, and improved small intestinal propulsion by 64%. Additionally, it downregulated serum NO (25%) and VIP (32%) while upregulating SP (49%) and 5-HT (78%) levels. Intestinal 5HTR4 and SERT expression increased by 78% and 71%, respectively, with MAOA suppression (25%). Microbial analysis revealed a 140% increase in *Dubosiella* and 49% in *Lactobacillus* abundance, alongside a 62% reduction in *Mucispirillum*. MBSFL contained polysaccharides (12.3% *w*/*w*) and organic acids, including hydroxy butyric acid (4.2 mg/mL). Conclusions: MBSFL alleviates constipation through dual mechanisms: modulating 5-HT pathway activity and restoring gut microbiota homeostasis.

## 1. Introduction

Constipation, which can be both a sign of underlying diseases and a disorder in itself, is influenced by various factors and poses challenges in achieving a steady state. Its clinical symptoms include irregular bowel movements, abdominal discomfort, and pain, impacting appetite and mood. Global data indicate that constipation affects approximately 18.9% of the elderly population [1], with higher prevalence observed among the elderly and women [2]. In specific regions of China, constipation is reported in 17.6% of people over 65 years [3], imposing a significant strain on healthcare resources [4]. Pharmacological remedies for constipation, such as stimulant laxatives and osmotic agents, can disrupt the balance of the intestinal microbiota, leading to potential health risks such as diarrhea, electrolyte imbalances, and increased susceptibility to infections. Moreover, the uniform dosing of these medications often fails to account for individual variations in patient physiology, disease severity, and response to treatment, which can result in suboptimal therapeutic outcomes and increased side effects. Consequently, addressing constipation through dietary interventions is increasingly advocated as a preferable approach.

5-HT is a critical neuromodulator in gastrointestinal motility, with over 95% synthesized by enterochromaffin cells in the gut. Its interaction with 5HTR_4_ stimulates enteric neurons and smooth muscle contraction, promoting peristalsis and accelerating colonic transit. Conversely, the SERT terminates 5-HT signaling via rapid reuptake, while MAO_A_ metabolizes 5-HT into inactive 5-hydroxyindoleacetic acid [5]. Dysregulation of this triad reduced 5HTR_4_ expression, SERT overexpression, or MAO_A_ hyperactivity disrupts 5-HT homeostasis, leading to impaired motility and constipation pathogenesis [6]. For instance, in slow-transit constipation, diminished 5HTR_4_ activation results in weakened colonic contractions, whereas elevated SERT activity reduces extracellular 5-HT availability [7]. Clinical studies further link MAO_A_ polymorphisms to altered 5-HT degradation rates and delayed gut transit. These findings underscore the centrality of 5-HT signaling in motility disorders and highlight therapeutic strategies targeting its regulatory axis.

The primary trigger for the onset of constipation is diminished intestinal motility, a process intricately linked to gut microbiota. Enhancing gut flora is pivotal in overcoming barriers to digestive motility. Studies have shown that wheat peptides can modulate the abundance of *Turicibacter*, *Bacteroides*, and *Streptococcus* at the genus level in the gut, leading to improved expression of proteins and genes that stimulate intestinal motility. Specifically, wheat peptides reduce the expression of AQP3 and ENaC subunits (β and γ), while promoting the expression of Claudin-1, ZO-1, HO-1, and NQO1. These changes improve water–salt metabolism and intestinal barrier function, and reduce oxidative stress, thereby mitigating constipation severity in mice [8]. Furthermore, a synbiotic combination of sucrose and *Latilactobacillus sakei* (Furu, 2019) was found to enhance beneficial gut bacteria populations, regulate intestinal flora, and alleviate constipation induced by diphenoxylate in mice [9]. Additionally, a hawthorn-probiotic postbiotic demonstrated efficacy in improving constipation in mice by upregulating *Lactobacillus plantarum* levels, modulating intestinal water and sodium metabolism, preserving intestinal barrier integrity, and balancing intestinal flora [10].

Beans are rich in dietary fibers, plant proteins, oligosaccharides, and isoflavones. Dietary fibers aid in promoting intestinal peristalsis and reducing fecal compactness [11]. Plant proteins and oligosaccharides serve as prebiotics, fostering the growth of beneficial gut flora and thereby balancing intestinal microbiota for optimal gut health [12]. Isoflavones exhibit antioxidant and anti-inflammatory properties, contributing to the mitigation of intestinal damage [13]. However, legumes often contain phytic acid and protease inhibitors that can impede nutrient absorption in the intestines. Excessive consumption of unprocessed legumes may lead to flatulence and indigestion [14].

Mung bean starch fermentation liquid (MBSFL) is a traditional fermented food in northern China, revered among the elderly for its health benefits. Studies have revealed that *Firmicutes* and *Proteobacteria* constitute over 94% of the total microbiota in MBSFL, with other prevalent bacteria including *Lactococcus*, *Acetobacter*, *Streptococcus*, and *Lactobacillus* [15].

MBSFL, a traditional beverage derived from the fermentation of mung bean starch, encapsulates the combined benefits of leguminous plants and the fermentation process, offering a multifaceted approach to alleviating constipation. The mung bean itself is a nutritional powerhouse, rich in dietary fiber, plant proteins, oligosaccharides, and isoflavones, which contribute to promoting intestinal peristalsis, reducing fecal compactness, and fostering beneficial gut flora [16,17]. The fermentation process further amplifies these benefits by enhancing the bioavailability of nutrients, increasing the content of bioactive compounds such as organic acids and polysaccharides [18], and introducing probiotics that can modulate the gut microbiome [19,20].

In this study, MBSFL was employed to investigate its alleviating effects on compound diphenoxylate-induced constipation in male Inbred Charles River (ICR) mice. Physiological indicators of constipation, such as body weight, fecal water content, time to pass the first black stool, and gastric emptying rate, were assessed. Serum indices, including SP, VIP, 5-HT, and NO, were measured. Histopathological analysis was conducted to evaluate changes in intestinal tissue microstructure. Gene expression levels of 5HTR_4_, MAO_A_, and SERT were examined, as these markers are central regulators of 5-HT bioavailability and signaling: 5HTR_4_ mediates 5-HT-induced smooth muscle contraction, SERT modulates 5-HT reuptake, and MAO_A_ governs its metabolic clearance. Their collective activity directly impacts gut motility and is influenced by microbial metabolites, bridging MBSFL’s prebiotic effects to 5-HT axis modulation. Additionally, differences in fecal microflora composition were analyzed to elucidate the mechanism by which MBSFL acts in the gastrointestinal tract to alleviate constipation.

## 2. Materials and Methods

### 2.1. MBSFL Preparation and Mass Spectrometry

Mung beans, sourced from Hebei, China, were initially cleaned and soaked in deionized water for 16 h. Following this, the beans were ground and homogenized using a flour mixing machine (Model JB-120, Jiubing, Shanghai, China). The resulting residue was filtered through an 80-mesh sieve to separate the supernatant. After allowing the mixture to settle, the supernatant was removed to isolate the mung bean starch. This washing process was repeated three times to ensure purity. The starch was then subjected to fermentation by *Lactiplantibacillus plantarum AR495* (CGMCC No. 23121, isolated from traditional fermented chili in Yunnan, China) at 32 °C for 48 h, followed by pasteurization at 60 °C for 45 min to obtain MBSFL.

The total polysaccharide content was quantified using the phenol–sulfuric acid colorimetric method [12]. The total acid content was determined in accordance with ISO750:1998, which specifies the method for determining titratable acidity in fruit and vegetable products. For organic acid analysis, MBSFL was diluted 10-fold with methanol and centrifuged at 10,000 rpm for 15 min. The supernatant was subsequently analyzed by mass spectrometry (Mass Spectrometer c3001, Qing PU Technology Ltd., Suzhou, China) to generate a master plot. Key peaks identified in the master plot were selected for secondary profiling, which involved comparing these peaks to standard references in the Massbank and Pub-Chem databases.

### 2.2. Animal Treatment and Experimental Design

This study was conducted in accordance with the guidelines of the Declaration of Helsinki and was approved by the Animal Ethics Committee of the Chinese Academy of Food and Fermentation Industry (Approval No. F610-2022-8, Approval Date: 10 March 2022).

Constipation Induction and Experimental Design: A total of 120 SPF-grade male ICR mice (Beijing Vitality Laboratory Animal Technology Co., Ltd., Beijing, China) were acclimatized for 5 days and divided into two cohorts.

**Figure 1:**
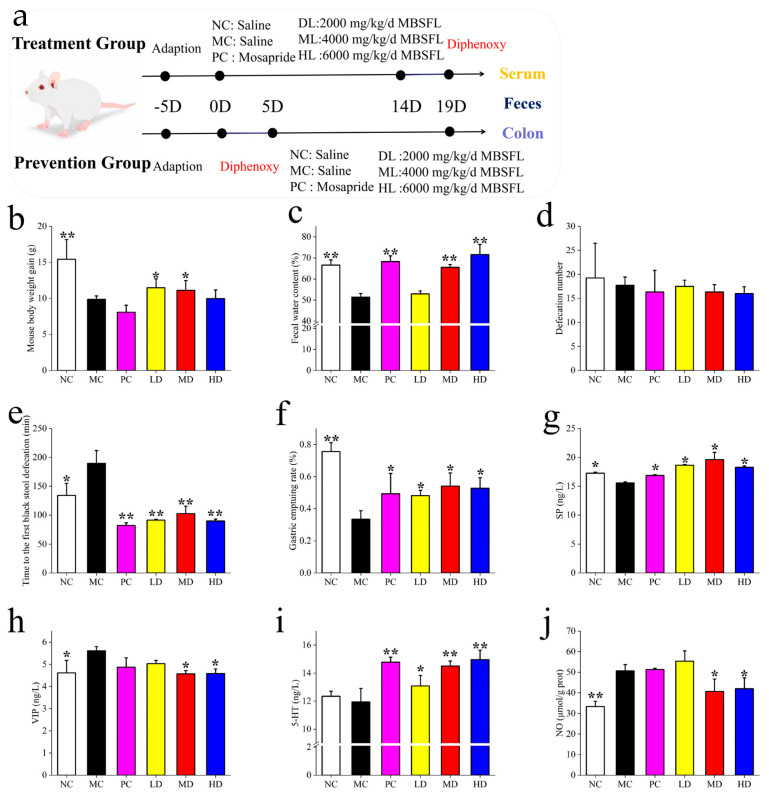
(**a**) Experimental design overview, (**b**) body weight gain, (**c**) fecal water content, (**d**) defecation frequency, (**e**) time to first black stool, (**f**) gastric propulsion rate, and serum levels of (**g**) SP, (**h**) VIP, (**i**) 5-HT, and (**j**) NO. Different groups include NC (blank control), MC (model control), PC (positive control), LD (low-dose MBSFL), MD (medium-dose MBSFL), and HD (high-dose MBSFL), n = 5~8. Significant differences are denoted by asterisks (*, **) as follows: * *p* < 0.05, ** *p* < 0.01.

Figure 1a shows the Treatment Group (n = 60), where constipation was induced by oral gavage of compound diphenoxylate (5 mg/kg bw/day) for 5 consecutive days, followed by a 14-day intervention.

Prevention Group (n = 60): Mice received compound diphenoxylate (5 mg/kg bw/day) simultaneously with interventions for 14 days. Each group was further divided into six subgroups (n = 10/subgroup):

Treatment Group: MC (saline, 0.9% NaCl), PC (positive control: 2 mg/kg bw mosapride citrate), LD (2000 mg/kg bw/day MBSFL), MD (4000 mg/kg bw/day MBSFL), and HD (6000 mg/kg bw/day MBSFL). Prevention Group: NC (normal control: saline, 0.9% NaCl), MC (model control: saline, 0.9% NaCl), PC (2 mg/kg bw mosapride citrate), LD (2000 mg/kg bw/day MBSFL), MD (4000 mg/kg bw/day MBSFL), and HD (6000 mg/kg bw/day MBSFL).

Administration Protocol, Diphenoxylate: Administered orally at 5 mg/kg bw/day (dissolved in saline) for 5 days (Treatment Group) or 14 days (Prevention Group). MBSFL and Controls: Delivered daily by oral gavage for 14 days. Total Study Duration: 19 days (5-day induction + 14-day intervention for the Treatment Group; 14-day co-administration for the Prevention Group).

Mice were housed under controlled conditions (temperature: 20 ± 2 °C; humidity: 50 ± 5%; 12 h light-dark cycle). Post-intervention, blood and fecal samples were collected after a 12 h fast, followed by euthanasia via cervical dislocation. Intestinal tissues (1 cm segment distal to the ileocecum) were harvested for analysis.

### 2.3. Bowel Movement Time and Weight

On the 20th day of the experiment, both groups underwent defecation testing. After 12 h of fasting, mice received an oral gavage of activated charcoal suspension (100 mg/mL) at a standardized dose of 2500 mg/kg bw (prepared by dissolving 10% activated charcoal in arabic gum solution, boiled until transparent, and adjusted to final volume with distilled water). Subsequently, mice were transferred to filter paper-lined cages. The latency to first fecal discharge and total fecal pellets within 5 h were recorded. Fecal samples were immediately collected in pre-weighed aluminum boxes for water content analysis.

Fresh fecal weight (M1) and dried weight (M2) were measured after dehydration at 110 °C for 4 h. Moisture content (%) was calculated as follows:

M1: weight of aluminum box containing fecal matter before drying;

M2: weight of aluminum box containing fecal matter after drying;

M3: weight of aluminum box after drying;

Moisture content of fecal (%) = [(M1 − M2)/(M1 − M3)] × 100%.

### 2.4. Gastric Emptying Rate and Small Intestinal Propulsion Rate

On the 21st day of the experiment, the mice were orally administered 0.5 mL of activated charcoal solution after a 12 h fasting period with no access to water. After 30 min, the mice were euthanized by decapitation, and their stomachs were immediately opened and weighed (m_1_). The stomachs were then immersed in ice water, cut open along the greater curvature, washed with saline, and reweighed as m_2_ to determine the gastric emptying rate. Subsequently, the small intestine was dissected, and the entire intestinal tube from the pylorus to the ileocecal region was carefully excised. The intestinal tube was straightened gently to ensure it was in a relaxed state, and the total length of the small intestine, as well as the distance from the pylorus to the most anterior end of the activated charcoal test propulsion, were measured. This information was used to calculate the propulsion rate of the small intestine for each mouse.

The gastric emptying rate was calculated using the formula: gastric emptying rate = ((m_1_ − m_2_)/m_1_) × 100%.

The small intestinal propulsion rate was determined using the formula: small intestinal propulsion rate = (length of activated charcoal test advancement/total small intestinal length) × 100%.

### 2.5. Serological Indicator

Blood samples were obtained from the eyeballs of the mice and left at room temperature for 4 h to allow for clotting. Subsequently, the samples were centrifuged at 4 °C and 3000 revolutions per minute for 10 min to separate the serum. The upper layer of the serum was carefully extracted for further analysis. The levels of enteric neurotransmitters, including 5-HT (ELISA Kit Cat# SEKSM-0016), NO (ELISA Kit Cat# BC1475), SP (ELISA Kit Cat# SEKSM-0040), and VIP (ELISA Kit Cat# SEKR-0112), were quantified in the serum using enzyme-linked immunosorbent assay (ELISA) kits purchased from Beijing Solarbio Science & Technology Co., Ltd. (Beijing, China), following the manufacturer’s instructions.

### 2.6. HE Staining of Intestinal Tissue

A 2 cm segment of the small intestine, located 2 cm from the ileocecal region, was collected and fixed in a 10% neutral formalin solution. Subsequently, paraffin sections were prepared and stained with hematoxylin–eosin to examine histological differences in the colon.

### 2.7. Western Blot Analysis

Proteins were extracted from the mouse’s small intestinal tissue using the Pierce™ RIPA Lysis Buffer Kit (Cat# 89900, Thermo Fisher Scientific, Waltham, MA, USA). The protein concentration was adjusted to 30 µg/mL using a BCA assay. Proteins were separated by sodium dodecyl sulfate–polyacrylamide gel electrophoresis (SDS-PAGE) with a 10% separating gel and 5% stacking gel (SDS-PAGE Gel Preparation Kit, Cat# P1200, Beijing Solarbio Science & Technology Co., Ltd., Beijing, China). After electrophoresis, the proteins were transferred to PVDF membranes (Cat# IPVH00010, Millipore, Burlington, MA, USA) and blocked with 5% skimmed milk powder (Cat# D8340, Beijing Solarbio Science & Technology Co., Ltd., China) for 1 h at room temperature. The membranes were incubated overnight at 4 °C with the following primary antibodies: 5-HT Receptor 4 (5HTR_4_) (Rabbit Polyclonal Antibody, Cat# 21165-1-AP, 1:1000 dilution) and Monoamine Oxidase A (MAOA) (Rabbit Polyclonal Antibody, Cat# 10539-178AP, 1:1000 dilution), both from Wuhan Sanying Biotechnology Co., Ltd. (Wuhan, China). After three washes with TBST buffer (Cat# T1082, Beijing Solarbio Science & Technology Co., Ltd., China), the membranes were incubated with HRP-conjugated goat anti-rabbit secondary antibody (Cat# 31460, Thermo Fisher Scientific, USA) for 1 h. Protein bands were visualized using the iBright™ FL1000 Imaging System (Thermo Fisher Scientific, USA).

### 2.8. 16S rRNA Amplicon Sequencing of the Microbiota

DNA was extracted and purified from mouse fecal samples using the TIANamp DNA Stool Kit Fecal Genomic DNA Extraction Kit (DP328-02, Tiangen Biochemical Science and Technology Company, Beijing, China) according to the instruction manual. Referring to previous research operations, the changes in bacterial flora under the region were detected by amplification of the V3–V4 variable region of the 16SrRNA gene, which was purified and quantified. Pyrophosphate sequencing was performed on the MiSeq platform using MiSeq Reagent Kit v3 (600 cycles in total), and raw data were processed and analyzed using the QIIME tool (Quantitative Insights Into Microbial Ecology 1.9.1, The Regents of the University of California, USA) after sequencing.

### 2.9. Statistical Analysis

Statistical analyses were performed using the SPSS 12.0 package (SPSS Inc., Chicago, IL, USA). Results were expressed using means ± SEM. Significance was analyzed using Pearson’s analysis, and correlation was analyzed using Spearman’s analysis, with *p* < 0.05 considered statistically significant. Graphing was performed using Origin 2022.

## 3. Results

### 3.1. MBSFL Component Analysis

The MBSFL was found to contain polysaccharide and organic acid components at concentrations of 16.8 ± 1.67 mg/mL and 11.09 ± 2.43 mg/mL, respectively. Mass spectrometry mapping was utilized to investigate the potential presence of hydroxybutyric acid, 3-hydroxypyruvic acid, benzoic acid, salicylic acid, uric acid, and octanoic acid in MBSFL.

### 3.2. Post-Induction Intervention (Treatment Modeling)

As shown in Figure 1b–j, the effects of MBSFL treatment on constipated mice were investigated by analyzing body weight gain, fecal water content, number of fecal pellets, and time to first black stool. Compared to the NC group, mice in the MC group showed significantly reduced weight gain, fecal water content, and gastric propulsion rate, along with an increased time to the first black stool, indicating successful construction of a constipation model using compound diphenoxylate. The PC, MD, and HD groups exhibited similar results in fecal water content, time to discharge the first black stool, and gastric propulsion rate. Fecal water content increased with MBSFL dose escalation, with significant improvements in gastric propulsion and reduction in time to the expulsion of the first black stool, akin to the effects of mosapride citrate. Notably, the PC group had lower body weight compared to the LD and MD groups, suggesting that while mosapride citrate relieved constipation, it did not address associated issues like decreased appetite and body weight, which MBSFL effectively mitigated. Interestingly, MBSFL did not impact the frequency of defecation across the groups.

In terms of intestinal peristalsis, changes in intestinal neurotransmitters were examined. The MC group exhibited significantly higher levels of VIP and NO compared to the NC group, with lower levels of SP. Following MBSFL intervention in the LD, MD, and HD groups, SP increased in the LD and MD groups, while VIP decreased in the MD and HD groups. Additionally, 5-HT increased and NO decreased in the MD and HD groups post-intervention.

**Figure 2:**
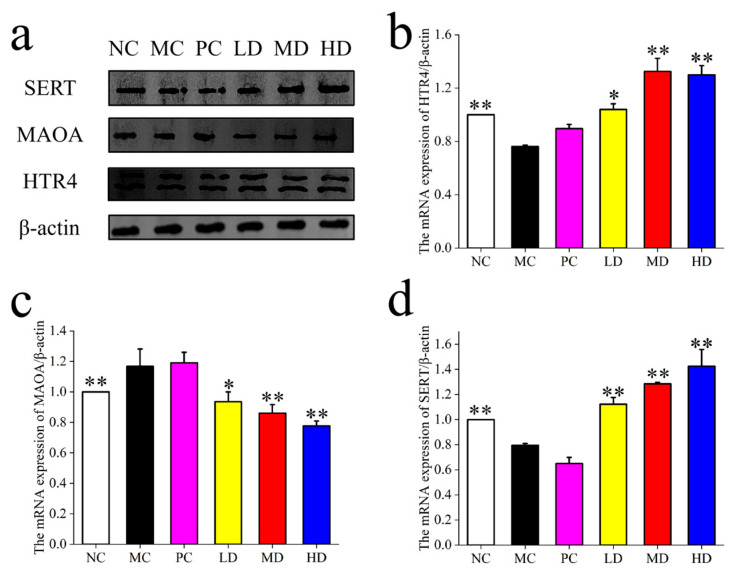
(**a**) The expression of 5HTR_4_, MAO_A_, and SERT (Treatment Groups), (**b**) 5HTR_4_; (**c**) MAO_A_, (**d**) SERT. Significant differences are denoted by asterisks (*, **) as follows: * *p* < 0.05, ** *p* < 0.01. Different groups include NC (blank control), MC (model control), PC (positive control), LD (low-dose MBSFL), MD (medium-dose MBSFL), and HD (high-dose MBSFL).

As shown in Figure 2, the expression of 5HTR_4_ and SERT was significantly higher in the LD, MD, and HD groups post-MBSFL intervention compared to the NC and PC groups, indicating a positive impact on intestinal neurotransmitter regulation. Conversely, the expression of MAO_A_ was significantly lower in the LD, MD, and HD groups compared to the NC and PC groups, suggesting that MBSFL intervention may reduce the degradation of 5-HT, thereby enhancing its availability and regulatory effects in the intestine.

**Figure 3:**
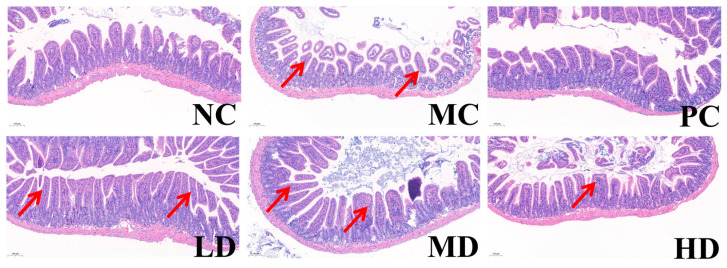
HE staining of the colon (treatment group). Noteworthy changes in the crypt furrow are indicated by red arrows. Different groups include NC (blank control), MC (model control), PC (positive control), LD (low-dose MBSFL), MD (medium-dose MBSFL), and HD (high-dose MBSFL).

As shown in Figure 3, microscopic analysis of colonic tissues revealed that the MC group exhibited tissue damage, while the PC group showed a well-organized tissue structure similar to the LD group. The MD and HD groups resembled the NC group, with improvements in tissue structure compared to the MC group. The LD group demonstrated the most effective relief of intestinal damage among the MBSFL doses.

Gut microbiota diversity was assessed using 16S rRNA gene sequencing. While the Shannon and Chao1 indices did not significantly differ between the MC and MBSFL groups, the Simpson index was lower in the MC group compared to the PC and LD groups. Changes in intestinal bacterial genera showed alterations in response to MBSFL intervention, with some genera positively correlated with intestinal neurotransmitter levels and negatively correlated with constipation-related parameters. Overall, these findings highlight the potential of MBSFL in modulating intestinal function, alleviating tissue damage, and influencing gut microbiota diversity in constipated mice.

**Figure 4:**
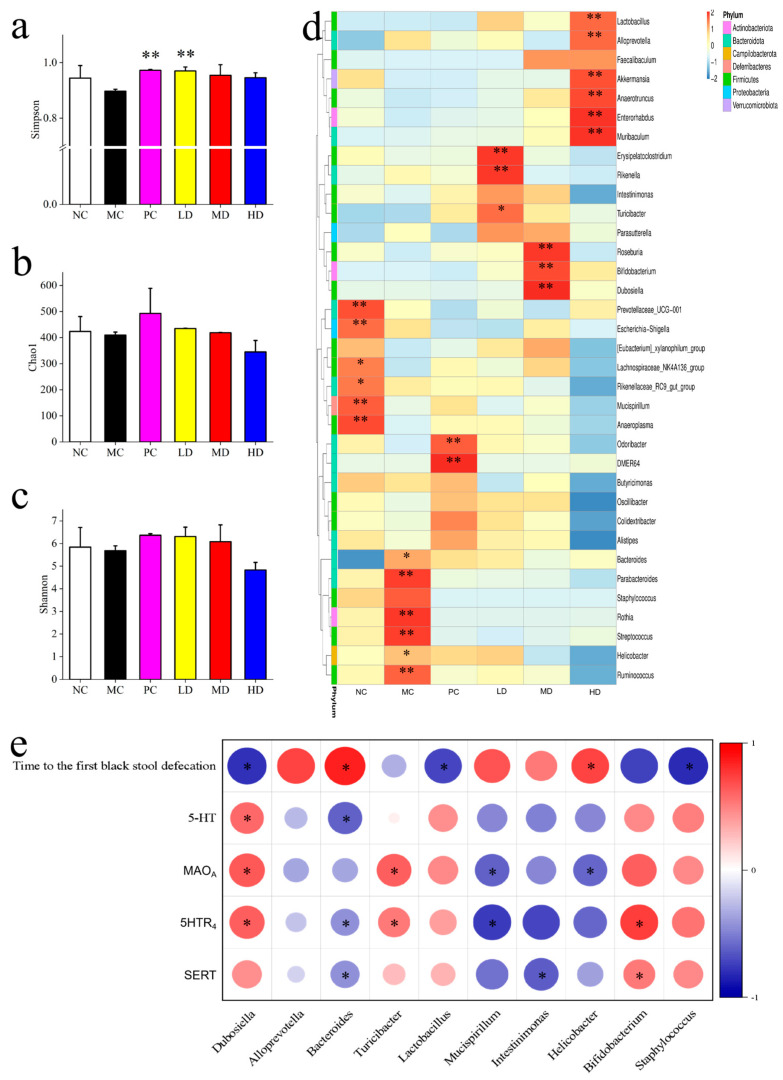
Gut microbial composition and correlations in the MBSFL Treatment Modeling. (**a**–**c**) display α-diversity metrics (Shannon, Chao1, Simpson). (**d**) shows a genus-level heat map of intestinal flora changes. (**e**) illustrates Spearman correlations between flora changes and constipation indicators, with blue and red circles indicating negative and positive correlations, respectively; circle size denotes correlation strength. Groups: NC (blank control), MC (model control), PC (positive control), LD (low-dose MBSFL), MD (medium-dose MBSFL), and HD (high-dose MBSFL). Significant differences are denoted by asterisks (*, **) as follows: * *p* < 0.05, ** *p* < 0.01.

Figure 4d depicts changes in the abundance of *Enterobacteriaceae* genera. In the MC group compared to the NC group, the abundance of *Prevotellaceae_UCG-001*, *Escherichia-Shigella*, *Lachnospiraceae_NK4A136_group*, and *Rikenellaceae_RC9_gut_group* decreased, while *Muribaculum* and *Anaeroplasma* decreased. Conversely, *Bacteroides*, *Parabacteroides*, *Rothia*, *Streptococcus*, *Helicobacter*, and *Ruminococcus* increased. Compared to the MC group, the PC group showed increased abundance of DEMR64 and *Odoribacter*; the LD group exhibited increases in *Turicibacter*, *Rikenella*, and *Erysipelatoclostridium*; the MD group had increased *Roseburia*, *Bifidobacterium*, and *Dubosiella*; and the HD group showed an increase in *Lactobacillus*, *Alloprevotella*, *Akkermansia*, *Anaerotruncus*, *Enterorhabdus*, and *Muribaculum*. Notably, more genera in the MC group than in the NC group were reduced by MBSFL intervention.

Spearman correlation analysis revealed interesting relationships between the mentioned genera and various parameters. Genera increased by MBSFL intervention, such as *Dubosiella*, *Turicibacter*, *Lactobacillus*, *Bifidobacterium*, and *Staphylococcus*, were positively correlated with SERT, 5HTR_4_, and MAO_A_ activity, as well as 5-HT serum levels (*p* < 0.05). Conversely, genera decreased by MBSFL, including *Bacteroides*, *Mucispirillum*, *Intestinimonas*, and *Helicobacter*, showed negative correlations with time to first black stool (*p* < 0.05).

### 3.3. Intervention Followed by Induction (Prevention Group)

**Figure 5:**
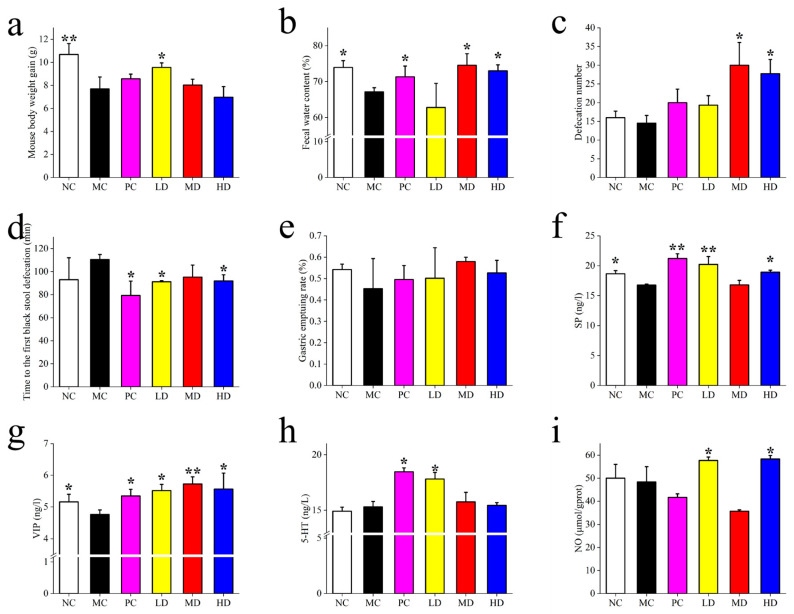
Depicts defecation, intestinal conditions, and serum levels in the Prevention Group, including (**a**) body weight gain, (**b**) fecal water content, (**c**) defecation frequency, (**d**) time to first black stool, (**e**) gastric propulsion rate, and serum levels of (**f**) SP, (**g**) VIP, (**h**) 5-HT, and (**i**) NO. Significant differences are denoted by asterisks (*, **) as follows: * *p* < 0.05, ** *p* < 0.01. Different groups include NC (blank control), MC (model control), PC (positive control), LD (low-dose MBSFL), MD (medium-dose MBSFL), and HD (high-dose MBSFL), n = 5~8.

Figure 5a–e illustrates the body weight condition, fecal parameters, and gastrointestinal functioning of mice in the Prevention Groups. Following the induction of constipation, mice in the MC group exhibited lower weight gain compared to the NC group, while the LD and MD groups effectively mitigated this decline. In terms of fecal water content, the MC group decreased by 9.25% relative to the NC group (*p* < 0.05), with the PC, MD, and HD groups showing significant improvements of 6.26%, 11.09%, and 8.75%, respectively, compared to the MC group (*p* < 0.05). The MD and HD groups displayed significantly higher numbers of bowel movements than the NC and MC groups (*p* < 0.05). Moreover, the time to first black stool was notably shorter by 16.97%, 13.80%, and 16.74% in the LD, MD, and HD groups compared to the MC group (*p* < 0.05).

Regarding serum biomarkers, the LD, MD, and HD groups exhibited significantly higher serum SP concentrations than the MC group (*p* < 0.05). Conversely, VIP concentrations were significantly lower in the DL and HL groups compared to the MC group (*p* < 0.05), while 5-HT levels were significantly higher in the LD and HD groups relative to the MC group (*p* < 0.05). Additionally, NO (nitric oxide) concentrations were significantly lower in the ML and HL groups compared to the MC group (*p* < 0.05).

**Figure 6:**
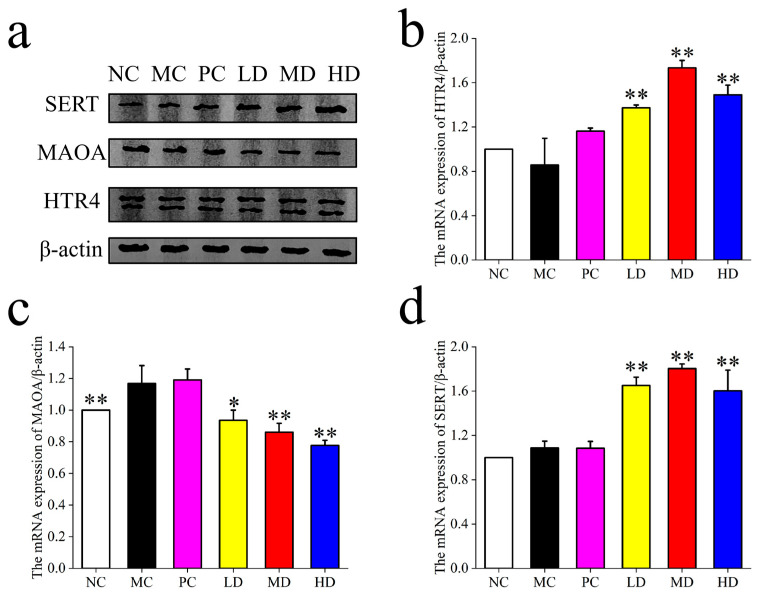
(**a**) The expression of 5HTR_4_, MAO_A_, and SERT (prophylactic groups), (**b**) 5HTR_4_; (**c**) MAO_A_, (**d**) SERT. Significant differences are denoted by asterisks (*, **) as follows: * *p* < 0.05, ** *p* < 0.01. Different groups include NC (blank control), MC (model control), PC (positive control), LD (low-dose MBSFL), MD (medium-dose MBSFL), and HD (high-dose MBSFL).

As shown in Figure 6, the expression of NC, MC was lower (*p* < 0.05), and 5HTR_4_ and SERT were significantly enhanced by the intervention of MBSFL (*p* < 0.05), while the expression of MAO_A_ was higher in the MC group (*p* < 0.05), and was reduced by the intervention of MBSFL, and it could be found that there was no reduction in the PC group (*p* < 0.05).

**Figure 7:**
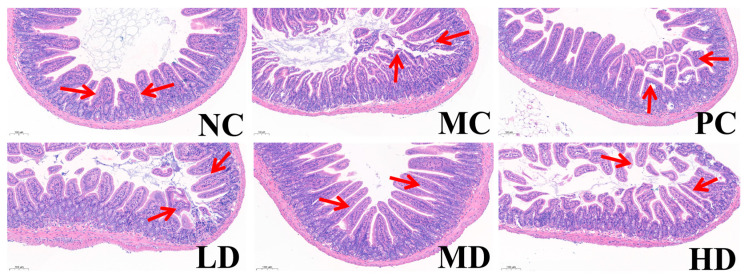
HE staining of the colon in the Prevention Group, showcasing notable changes in the crypt furrow, with distinct group labels and scale bar reference. Different groups include NC (blank control), MC (model control), PC (positive control), LD (low-dose MBSFL), MD (medium-dose MBSFL), and HD (high-dose MBSFL). The area indicated by the red arrow shows that the hidden groove is relatively large.

Microscopic examination of colonic tissues in the Prevention Group (Figure 7) revealed that the NC, PC, LD, and MD groups exhibited uniform tissue structure with fewer and shallower crypts compared to the MC group. These observations suggest that MBSFL can prevent colon damage induced by the compound diphenoxylate. Notably, the preventive effect of the PC group was less pronounced compared to the MD group, indicating that mosapride citrate may have limited efficacy in alleviating intestinal damage in the preventive model.

**Figure 8:**
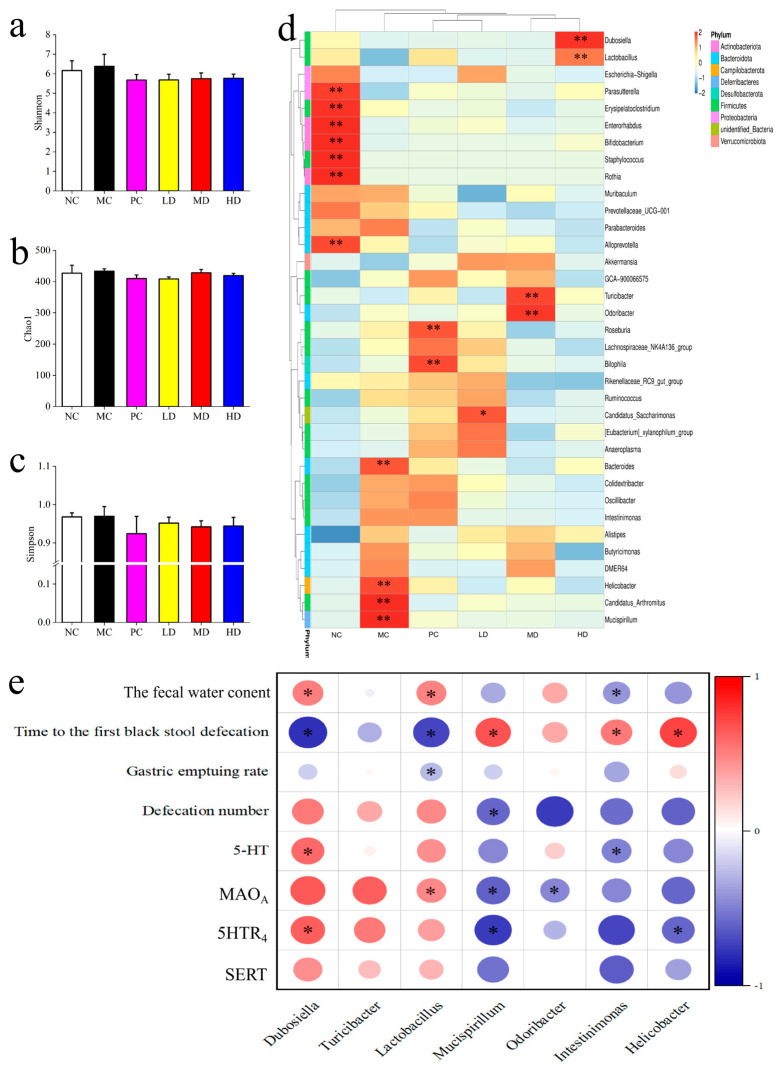
Gut microbial composition and correlations in the MBSFL Prevention Group. (**a**–**c**) display α-diversity metrics (Shannon, Chao1, and Simpson). (**d**) shows a genus-level heat map of intestinal flora changes. (**e**) illustrates Spearman correlations between flora changes and constipation indicators, with blue and red circles indicating negative and positive correlations, respectively; circle size denotes correlation strength. Groups: NC (blank control), MC (model control), PC (positive control), LD (low-dose MBSFL), MD (medium-dose MBSFL), and HD (high-dose MBSFL). Significant differences are denoted by asterisks (*, **) as follows: * *p* < 0.05, ** *p* < 0.01.

As shown in Figure 8, in the preventive group, the impact of MBSFL on the intestinal flora diversity and homogeneity in mice did not show significant differences, which is consistent with the findings in the Treatment Group. Several genera, including *Parasutterella*, *Erysipelatoclostridium*, *Enterorhadus*, *Bifidobacterium*, *Staphylococcus*, *Alloprevotella*, and *Rothia*, exhibited reduced abundance (*p* < 0.05) in the MC group compared to the NC group, while *Mucispirillum*, *Candidatus-Arthromitus*, *Helicobacter*, and *Bacteroides* showed increased levels (*p* < 0.05). Following MBSFL intervention, there was a notable decrease in *Mucispirillum* and *Candidatus-Arthromitus* compared to the MC group, with a more pronounced effect than in the NC group. Furthermore, *Helicobacter* and *Bacteroides* levels were reduced (*p* < 0.05), while the proportion of *Candidatus_Saccharimonas* increased (*p* < 0.05) in the LD group; *Turicibacter* and *Odoribacter* increased in the MD group; and *Dubosiella* and *Lactobacillus* increased (*p* < 0.05) in the HD group.

The abundance of *Turicibacter* and *Dubosiella* increased following MBSFL intervention, exhibited positive correlations with the expression of SERT, 5HTR_4_, and MAO_A_, as well as with the number of defecations and fecal water content (*p* < 0.05), and a negative correlation with the time to the first black stool. Conversely, the abundance of *Mucispirillum*, *Odoribacter*, *Intestinimonas*, and *Helicobacter* decreased following MBSFL intervention, exhibited negative correlations with the expression of SERT, 5HTR_4_, and MAO_A_, as well as with the number of defecations and fecal water content (*p* < 0.05), and a positive correlation with the time to the first black stool *(p* < 0.05).

## 4. Discussion

The potential benefit of MBSFL lies in its ability to integrally modulate intestinal hormones, thereby positively affecting intestinal motility. Our results showed that MBSFL significantly improved intestinal transport in a mouse model of constipation induced by cotrimoxazole by modulating the levels of SP, VIP, 5-HT, and NO. SP is a neuropeptide that promotes intestinal peristalsis. Increased levels of SP correlate with the induction of intestinal motility [21]. By increasing the levels of SP, MBSFL may stimulate intestinal peristalsis and facilitate the propulsion of intestinal contents. VIP is known to inhibit intestinal motility [22]. By decreasing the levels of VIP, MBSFL may reduce VIP-induced inhibition of intestinal motility, which may be beneficial in the treatment of constipation. 5-HT is one of the most potent enhancers/stimulators of intestinal motility, exerting its effects through the activation of the 5-HT4 receptor. MBSFL, by increasing the level of 5-HT and/or upregulating the expression of the 5HTR_4_, may enhance intestinal propulsive activity [23]. NO plays a dual role in intestinal peristalsis [24,25], as it can both promote and inhibit intestinal mobility. Our findings showed that MBSFL treatment led to a significant increase in NO levels in the serum of mice with cotrimoxazole-induced constipation. This increase in NO levels suggests that MBSFL may enhance the promoting effects of NO on intestinal peristalsis, thereby optimizing intestinal motility functions. Specifically, MBSFL may act by influencing the interactions between these hormones. For example, MBSFL may have enhanced the promoting effects of SP and 5-HT while decreasing the inhibitory effects of VIP and modulating the levels of NO, thereby integrally regulating intestinal motility.

The regulatory effects of MBSFL are not limited to the intestinal hormone levels but also involve the metabolism and transport mechanisms of 5-HT. 5-HT plays a key role in intestinal motility, and changes in its level directly affect intestinal motility function. 5-HT can promote intestinal motility through its binding to the 5HTR_4_ receptor [23], but its effects are limited due to activation of SERT, a transporter that decreases the bioavailability of 5-HT at the receptor site. So it may be concluded that 5HTR_4_ and SERT may have opposite roles in the regulation of 5-HT concentrations [26]. MAO_A_, by metabolizing 5-HT, is also involved in the regulation of 5-HT levels [27,28]. In constipation, the expression and activity of these molecules may be altered, affecting intestinal peristalsis and secretory function [29], leading to constipation. Thus, it is known that the action of MBSFL enhances the metabolism of 5-HT and increases the efficiency of utilization, restoring normal intestinal motility. As in the present study, *Lactiplantibacillus plantarum AR495* modulated the expression of 5-HT and 5HTR_4_ and alleviated colonic transit hyperactivity [30]. The paradoxical increase in both 5-HT bioavailability and SERT expression observed in this study can be attributed to gut microbiota-mediated regulation of 5-HT homeostasis. Microbial metabolites enhance EC cell-derived 5-HT synthesis, overriding SERT-mediated clearance capacity. MAOA downregulation prolongs 5-HT signaling duration, compensating for SERT upregulation by reducing metabolic degradation. SERT overexpression may reflect adaptive feedback to elevated 5-HT, as microbial metabolites can modulate SERT membrane trafficking without altering intrinsic transporter activity.

Constipation is often associated with intestinal dysbiosis, a disruption in the balance of beneficial bacteria. Our results suggest that MBSFL effectively modulates intestinal flora, potentially restoring the balance of beneficial bacteria without disrupting the microbial equilibrium, in both therapeutic and preventive models [31]. This modulation may contribute to the mitigation of constipation by influencing the gut microbiota composition.

Patients with constipation often exhibit disturbances in their intestinal flora, characterized by significant differences in the diversity and structure of colonic mucosal flora compared to healthy individuals. Specifically, there is a notable decrease in the abundance of *Prevotella* and a corresponding increase in the abundance of *Firmicutes*. Additionally, the populations of beneficial bacteria such as *Bifidobacterium*, *Lactobacillus*, and *Anaplasmaspp*. are significantly reduced, while potentially pathogenic bacteria, including *Escherichia coli*, *Staphylococcus aureus*, and members of the *Enterobacteriaceae* family, are significantly elevated. These findings align with previous studies indicating a close association between specific bacterial populations and constipation. For instance, *Bacteroides*, *Roseburia*, and *Coprococcus 3* have been linked to a reduced abundance of beneficial bacteria [32], and risky flora such as *Ruminococcaceae UCG005*, *Eubacterium nodatum* group, *Butyricimonas*, and *Bacteroidetes* are associated with an increased risk of constipation. A significantly higher abundance of *Clostridium* has also been correlated with constipation [33,34], providing a rationale for the relief of constipation through modulation of MBSFL-affected flora.

Moreover, our observations indicate that the effects of MBSFL on gut flora are influenced by the method of ingestion. This suggests that the initial state of the gut flora plays a crucial role in determining the outcome. For example, in the approach where modeling is followed by intervention, the gut flora may have already been altered by the disease model, which could affect the initial state of the flora and subsequently influence the efficacy of the fermentation fluid. Conversely, the intervention followed by a modeling approach may allow for adjustments to the flora before it is impacted by the disease state, potentially leading to different outcomes. To illustrate this concept, consider the example of water intake before and after alcohol consumption. Drinking a large amount of water before alcohol consumption has limited effects on preventing hangover symptoms. However, drinking a large amount of water after alcohol consumption can significantly alleviate the discomfort associated with hangovers [35]. This example highlights the importance of the timing and method of intervention in modulating the body’s response to a stressor, similar to how the method of MBSFL ingestion can influence its effects on gut flora.

The composition of intestinal flora is closely linked to disease markers and therapeutic outcomes. Our Spearman correlation analysis revealed that MBSFL administration leads to significant changes in intestinal flora composition mediated through microbial metabolites such as SCFAs. As highlighted in our results, lactic acid bacteria (LAB) fermentation induces enzymatic hydrolysis of starch, preferentially targeting high-molecular-weight polymers. This process generates low-molecular-weight oligosaccharides and monosaccharides, which are subsequently metabolized by LAB into organic acids (e.g., lactate and acetate). The presence of these acids—absent in native starch—serves as indirect evidence of starch depolymerization and microbial utilization. In the context of MBSFL intervention, the accumulation of LAB-derived organic acids and residual hydrolysates likely synergizes with endogenous microbial metabolites to enhance bioactivity. Organic acids lower colonic pH, selectively promoting the growth of SCFA-producing bacteria while suppressing pathobionts like Enterobacteriaceae, thereby rectifying the Firmicutes/Bacteroidetes imbalance observed in constipation. The combined action of LAB-derived acids and microbial SCFAs amplifies 5-HT_4_ receptor responsiveness, synergizing with the observed MAO_A_ downregulation to prolong prokinetic signaling. For example, the increased abundance of *Dubosiella* and *Lactobacillus* likely enhances SCFA production, which directly stimulates 5-HT synthesis in enterochromaffin cells via free fatty acid receptor 3 activation. Concurrently, the 62% reduction in *Mucispirillum* may alleviate intestinal inflammation, thereby improving 5HTR_4_ sensitivity [35]. These microbial shifts correlate with a 78% increase in 5HTR_4_ expression, a 71% increase in SERT expression, and a 25% decrease in MAO_A_ expression. The causal relationship is supported by three mechanisms: 1. Microbial SCFAs enhance 5-HT bioavailability: Butyrate upregulates tryptophan hydroxylase in EC cells, overriding SERT-mediated clearance [20]. 2. MAO_A_ suppression: Reduced MAOA activity prolongs 5-HT signaling duration, counterbalancing SERT upregulation [36,37]. 3. Mucosal barrier restoration: *Lactobacillus*-derived metabolites strengthen tight junctions, reducing systemic endotoxin leakage and secondary inflammation that impairs motility [38].

The intervention approach for MBSFL is more favored over prevention. The reason is that compared to the positive control group, MBSFL’s first intervention will be more prominent in its effectiveness, respectively, in terms of the relief of weight loss due to constipation, the restoration of fecal water content, and the alleviation of intestinal damage. It is more evident that MBSFL, as a food, has minimal side effects, considering that the deeply processed starch, in addition to being able to demonstrate functional activity, also has a certain nutritional value, which cannot be replaced by positive drugs.

## 5. Conclusions

MBSFL alleviates constipation in mice by modulating the gut microbiota-5HT axis: it enhances intestinal motility via upregulating 5HTR_4_ and downregulating SERT expression, while restoring microbial diversity to favor short-chain fatty acid-producing genera (e.g., *Lactobacillus* and *Bifidobacterium*). These findings position MBSFL as a diet-derived prebiotic candidate for preventing gut dysmotility disorders.

## Data Availability

The original contributions presented in this study are included in the article. Further inquiries can be directed to the corresponding author.

## Abbreviations

The following abbreviations are used in this manuscript:

MBSFL	Mung bean starch fermentation liquid
SP	Substance P
VIP	Vasoactive intestinal peptide
5-HT	5-Hydroxytryptamine
NO	Nitric oxide
5HTR_4_	5-Hydroxytryptamine receptor 4
MAO_A_	Monoamine oxidase A
NC	Normal control group
MC	Model control group
PC	Positive control group
LD	Low dose group
MD	Medium dose group
HD	High dose group
